# Proinflammatory and Anti-Inflammatory Cytokines Mediated by NF-*κ*B Factor as Prognostic Markers in Mammary Tumors

**DOI:** 10.1155/2016/9512743

**Published:** 2016-02-16

**Authors:** Gustavo Rodrigues Martins, Gabriela Bottaro Gelaleti, Marina Gobbe Moschetta, Larissa Bazela Maschio-Signorini, Debora Ap. Pires de Campos Zuccari

**Affiliations:** ^1^Laboratório de Investigação Molecular no Câncer (LIMC), Faculdade de Medicina de São José do Rio Preto (FAMERP), Department of Molecular Biology, Graduate Program in Health Science, Avenida Brigadeiro Faria Lima 5416, Vila São Pedro, 15090-000 São José do Rio Preto, SP, Brazil; ^2^Laboratório de Investigação Molecular no Câncer (LIMC), Faculdade de Medicina de São José do Rio Preto (FAMERP), Department of Biology, Graduate Program in Genetics, Universidade Estadual Paulista “Júlio de Mesquita Filho” (UNESP/IBILCE), Rua Cristóvão Colombo 2265, Jardim Nazareth, 15054-000 São José do Rio Preto, SP, Brazil; ^3^Laboratório de Investigação Molecular no Câncer (LIMC), Faculdade de Medicina de São José do Rio Preto (FAMERP), Avenida Brigadeiro Faria Lima 5416, Vila São Pedro, 15.090-000 São José do Rio Preto, SP, Brazil

## Abstract

Inflammation results in the production of cytokines, such as interleukin- (IL-) 4 and IL-10 with immunosuppressive properties or IL-6 and TNF-*α* with procarcinogenic activity. Furthermore, NF-*κ*B is the major link between inflammation and tumorigenesis. This study verified the interaction between active inflammatory cytokines in the tumor microenvironment and serum of female dogs with mammary tumors and their correlation with the clinicopathological characteristics and overall survival. Measurement of gene expression was performed by qPCR and protein levels by ELISA/Luminex. High gene and protein expression levels of NF-*κ*B, IL-6, and TNF-*α* were found in association with characteristics that reflect worse prognosis and a negative correlation between TNF-*α* protein expression and survival time was observed (*p* < 0.05). In contrast, high gene and protein expression levels of IL-4 and IL-10 were associated with characteristics of better prognosis and an increased level of IL-4 and a longer survival time of animals were obtained (*p* < 0.05). In addition, there was a positive correlation between TNF-*α* and IL-6 expression in association with NF-*κ*B. The results show a significant correlation of these cytokines with tumor development, associated with NF-*κ*B expression and cytokines promodulation, showing that these biological factors could be used as predictive and prognostic markers in breast cancer.

## 1. Introduction

The microenvironment is a dynamic system that is largely composed of inflammatory cells and cytokines, including tumor cells and stroma (the latter of which consists of immune cells and the surrounding extracellular matrix) [1, 2]. Under specific stimulation conditions, inflammation can result in the production of antiangiogenic cytokines, which suppress tumor growth. Despite being antagonistic to the action of tumor suppression, in most tumors, inflammatory cells can promote angiogenesis, growth, tumor cell migration, tissue degradation, and remodeling steps that are characteristic of the chronic inflammation associated with intense tissue disorders typical of acute inflammation [1, 3–7].

The transcription factor nuclear kappa B (NF-*κ*B) plays a major role in innate immunity and inflammatory responses [8] and it is considered the major link between inflammation and tumorigenesis [1]. This factor activates antiapoptotic genes [9–11] and could promote the transcription of genes associated with growth, invasion, and metastasis, being the key factor that allows precancerous and malignant cells to escape apoptosis [1].

Interleukins (ILs) could stimulate many signaling pathways and thus regulate the transcription of target genes involved in cellular proliferation and survival [12]. Besides that, they act as immune mediators [13] and have been linked to cancer, playing a key role in the recruitment and positioning of leukocytes in carcinogenesis [14]. The recruitment and activation of immune cells and the antitumor effects of IL-4, an anti-inflammatory cytokine, are involved in the inhibition of angiogenesis [15], inflammation, thrombosis, growth, and invasion in some cancers [16, 17]. In addition to these effects on stromal cells, some studies have reported that IL-4 has antitumor functions by inducing apoptosis in several types of tumor cells including breast cancer, renal cell carcinoma, and hepatocellular carcinoma [18].

IL-10 is a cytokine produced by Th1 and Th2 cells [19] as well as by tumor cells and has potent immunosuppressive properties [20]. Originally known as an inhibitor factor of the synthesis of cytokines [7], IL-10 suppresses the production of IL-1*α*, IL-1*β*, tumor necrosis factor- (TNF-) *α*, IL-6, IL-8, IL-12, IL-18, granulocytes, inflammatory macrophages, and NF-*κ*B [7, 21, 22]. This cytokine acts concurrently with IL-3 and IL-4 to stimulate the proliferation of mast cells, peripheral blood lymphocytes [7], and macrophages, and their anti-inflammatory action has been suggested in tumor microenvironment [22].

On the other hand, IL-6 has strong procarcinogenic activity due to its role in tumor cell proliferation, survival, angiogenesis, inflammation, and metastasis [7, 23]. In addition, IL-6 is one of the activation signals for NF-*κ*B, promoting cell differentiation and subsequent metastasis [24], thus reinforcing the close link between inflammatory mediators and neoplastic progression [1]. Therefore, TNF-*α* induces proinflammatory cytokines such as IL-1 [25] and IL-6 [26, 27], during the inflammatory response [26–28]. The interaction of this cytokine with its receptor (TNFR) induces apoptotic and antiapoptotic effects in different cell systems [11], and it can promote cell proliferation and inhibits apoptosis in the tumor microenvironment [29], at least indirectly, through the induction of NF-*κ*B [26].

A better understanding of the complexity of these biological factors' network, together with the characterization of new genes, aids in uncovering the molecular mechanisms of inflammatory diseases and cancer [30]. In this way, the objective of this work was to ascertain the role of anti-inflammatory cytokines, IL-4 and IL-10, and proinflammatory cytokines, IL-6 and TNF-*α*, in mammary tumors of female dogs and to correlate these results with the gene and protein expression of NF-*κ*B. In addition, we sought to verify the correlation of these cytokines with clinicopathological parameters, cancer progression, and overall survival, to determine their prognostic value in mammary tumors.

## 2. Material and Methods

### 2.1. Sample Processing

Peripheral blood and tumor samples were obtained from 30 female dogs with malignant mammary neoplasia (test group) attended to in São José do Rio Preto, SP, Brazil, veterinary clinics in the years 2011 and 2012. Animals with mammary tumors were included regardless of breed, weight, or age. These animals were followed up for 540 days after tumor excision and blood collection. For control group, peripheral blood samples from 30 female dogs and five samples of mammary tissue adjacent to mammary cancer were obtained from animals that underwent elective hysterectomy as a preventive measure, after completion of the consent form by their owners. The criteria for the control group were rigorously followed and included animals outside the estrus period, animals with no tumor history, and animals with no detectable disease/inflammation in the period or surgery in the next study period. In addition, animals that died of causes other than tumor recurrence or metastasis were excluded from the study.

Peripheral blood (3 mL) was collected in a CORVAC serum separator tube (Labor Import, São Paulo, SP), stored at 4°C, processed by centrifugation (1000 ×g, 25 min), and cryopreserved at −80°C for posterior Enzyme Linked Immunosorbent Assay (ELISA) and xMAP protein analysis. Tumor samples were collected and divided into two pieces. The first one was fixed in 10% formaldehyde buffer for 24 hours and paraffin embedded for histopathology classification after hematoxylin-and-eosin (HE) stain. Tumor grading was ascertained according to Misdorp et al. [31] by the Armed Forces Institute of Pathology (AFIP). The second piece was collected in a Falcon tube containing the RNA-stabilizing solution, RNAlater (Life Technologies®), stored at room temperature for 24 hours, and posteriorly used for RNA extraction and qPCR analysis.

### 2.2. Clinicopathological Characteristics

Female dogs from the test group were evaluated with respect to physical (age), pathological (time course (interval between tumor diagnosis and surgical removal), tumor nodules location, lymph node involvement, tumor mass size, clinical staging, ulceration, and vascularization), and clinical (metastasis, local recurrence, and death) characteristics (Table 1). The parameters employed for the classification of clinical tumor grading were in accordance with the TNM (size, lymph node involvement, and metastasis) system established by the World Health Organization (WHO) for canine mammary gland tumors [32]. The clinical grade was assigned as I, II, III, or IV according to the extent of the tumor and the prognosis.

The age of the animals varied from 7 to 14 years (mean = 10 years) and there was predominance of Poodle (19%) and Dachshund (24%) races. In terms of the clinicopathological characteristics, most animals had a tumor for more than six months (54%) and were of clinical stage III or IV (54%). The proportions of animals with lymph node involvement, animals with metastasis/local recurrence, or animals that died during the study were 16%, 27%, and 27%, respectively (Table 1).

### 2.3. Gene Expression by Quantitative RT-PCR (qPCR)

For total RNA extraction, the tumor samples were manually processed into smaller pieces (100 mg/piece), and the pieces were then immersed in TRIzol reagent (Invitrogen Life Technologies®) and macerated with a Politron (Dremel, Mexico). Previously, tumor samples were treated with DNase and 10x buffer (10 mM Tris-HCl, pH 7.5; 50 mM CaCl_2_; and 10 mM MgCl_2_) (RNeasy Mini Kit, Qiagen) followed by the addition of ethylenediaminetetraacetic acid (EDTA) to deactivate or eliminate proteins binding to template DNA.

The cDNA was synthesized using a High Capacity cDNA kit (Applied Biosystems®). The qPCR was performed in triplicate using the StepOne Plus Real Time PCR system (Applied Biosystems) and TaqMan Universal Master Mix (Applied Biosystems). The following specific primers were used:* NF-κB*, Cf02622551_m1 (Applied Biosystems);* IL-4*, Cf02623112_m1 (Applied Biosystems);* IL-6*, Cf02624153_m1 (Applied Biosystems);* IL-10*, Cf02624265_m1 (Applied Biosystems); and* TNF-α*, Cf02628236_m1 (Applied Biosystems).

The following qPCR conditions were used: an initial step of 2 minutes at 50°C; denaturation for 10 minutes at 95°C; 40 cycles at 95°C for 15 seconds and 60°C for one minute to anneal primers and extend the chains, respectively; and 35 seconds at 65°C to collect the signal. The dissociation curve was generated after amplification and included a step of 15 seconds at 95°C followed by 1 min at 60°C. Each transcript level was normalized to the expression of* RPS19* (sense (5′-GCC TTC CTC AAA AAG TCT GGG-3′), antisense (5′-GCT TGC TCC CTA CGA TGA GAA C-3′), and probe (5′-CCC TGA ATG GGT GGA C-3′) (Applied Biosystems)) and* RPL8,* Cf02663820_m1 (Applied Biosystems), which were used as endogenous control genes.

The relative quantification (RQ) value of the expression of interest genes was determined with DataAssist v3.0, by the quantification method related to the average of the normalizing genes used as endogenous controls (ΔΔCt) [33] and showed by Log_10_. The gene expression of each sample was separately analyzed and classified as underexpressed (samples giving <0 Log_10_ measurement) and overexpressed (samples with quantification >0 Log_10_). The samples were tested in triplicate and all experiments included negative controls.

### 2.4. Protein Expression by ELISA and xMAP Protein Assays

NF-*κ*B (dog nuclear factor (NF) kappa B p105 subunit (NF-*κ*B1), CUSABIO Biotech Co., Hubei, China) and IL-4 (USCN Life Science Inc., Houston, TX, USA) protein expression analysis was performed according to the manufacturers recommendations. First of all, the plate was precoated with a specific antibody for NF-*κ*B and IL-4. Then, the standards and samples were added in each well and incubated for 2 hours at 37°C. A biotin-conjugated antibody and a horseradish peroxidase- (HRP-) avidin conjugate were added to the plate to amplify the signal and to increase the detectability of the target molecule. A chromogenic substrate (3-3-5-5-tetramethylbenzidine, TMB) was then added to detect the antibody/analyte binding in the reaction. Finally, sulfuric acid (stop solution) was added to stabilize the color development to enable accurate measurement of the intensity at 450 nm.

The analysis of IL-6, IL-10, and TNF-*α* proteins was performed using the MILLIPLEX MAP Kit (CCYTOMAG-90K; Millipore Corporation, USA), and the protein levels were analyzed using the Luminex xMAP MAGPIX (Millipore Corporation, USA). The standard, control, and samples were added to the appropriate wells as were magnetic beads coated with specific antibodies for IL-6, IL-10, and TNF-*α*. The plate was sealed and incubated overnight at 4°C with agitation on a plate shaker. The detecting antibody was added, and the plate was incubated. Streptavidin-phycoerythrin was added to each well to detect antibody/analyte binding. Measurements were performed at 635 nm to excite the magnetic beads and 525 nm to detect phycoerythrin.

The reaction intensity was proportional to the concentration of the cytokines and NF-*κ*B. The calculation of OD was determined through the adjustment curve four-parameter logistic (4-PL) using the SkanIt software for the Multiskan FC 2.5.1.

### 2.5. Statistical Analysis

The results were previously subjected to descriptive analyses for the determination of normality. Clinicopathological characteristics, gene expression, and protein quantification were analyzed in the test and control groups, and the differences between these groups were determined by Student's *t*-test or ANOVA followed by the Bonferroni test. All values are expressed as the mean ± standard deviation (SD).

The cutoff points for the protein analyses were established by the receiver operating characteristic (ROC) curve. For the ROC curve, the protein expression levels from female dogs that died were compared with those that survived until the end of the followup period. Survival curves were plotted by the Kaplan-Meier method and the differences between the curves were evaluated by a log-rank test and hazard function.

Spearman's rank correlation analysis was performed to describe the correlation between proinflammatory and anti-inflammatory cytokines protein and gene expression with NF-*κ*B. In addition, the mutual relationship between proinflammatory and anti-inflammatory cytokines was analyzed. A multivariate logistic regression analysis was employed to evaluate the simultaneous influence of the prognostic factors on animal death. A *p* value <0.05 was considered statistically significant. All analyses were performed using GraphPad Prism 4 and StatsDirect software.

## 3. Results

### 3.1. Differential Gene and Protein Expression of* NF-κB*,* IL-4*,* IL-10*,* IL-6*, and* TNF-α* Associated with Clinicopathological Features and Survival

**Figure 1:**
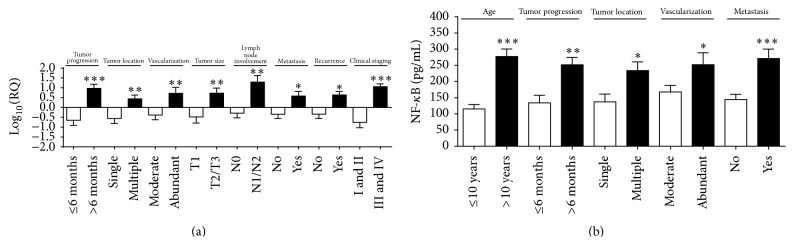
*NF-κB* gene overexpression correlated with (a) tumor progression greater than 6 months; multiple tumor locations; abundant tumor vascularization; tumor mass size greater than 3 cm; lymph node involvement; presence of metastasis; recurrence and clinical staging III or IV. Higher NF-*κ*B protein levels correlated with (b) with age > 10 years; tumor progression greater than 6 months; multiple tumor locations; abundant tumor vascularization; and presence of metastasis. Error bars ± standard error; ^*∗*^ *p* < 0.05; ^*∗∗*^ *p* < 0.01; ^*∗∗∗*^ *p* < 0.001 indicate significant differences according to Student's *t*-test.

The* NF-κB* gene was significantly overexpressed in animals with tumor time course greater than six months (*p* < 0.0001); multiple tumor locations (*p* = 0.005); abundant tumor vascularization (*p* = 0.009); tumor mass size greater than three centimeters (cm) (*p* = 0.005); lymph node involvement (*p* = 0.008); metastasis (*p* = 0.02); recurrence (*p* = 0.01); and clinical stage III or IV (*p* < 0.0001) (Figure 1(a)).

In the same way, the NF-*κ*B protein expression was significantly higher in animals older than 10 years (*p* = 0.0001), as well as in animals with a tumor time course of less than six months (*p* = 0.001), multiple tumor locations (*p* = 0.01), abundant tumor vascularization (*p* = 0.03), and metastasis (*p* = 0.0005) (Figure 1(b)). The best cutoff point for NF-*κ*B established by the ROC curve to discriminate the high risk of death was 219.5 pg/mL (sensitivity (95% CI) = 71%, specificity (95% CI) = 41%). No correlation was observed between NF-*κ*B serum levels and survival time (OR 2.003; 95% CI = 0.5553 to 8.983; *p* = 0.29) (data not shown).

**Figure 2:**
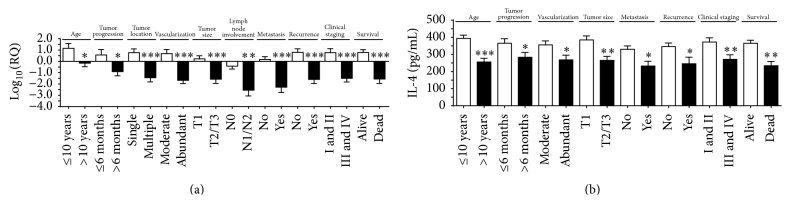
*IL-4* gene overexpression correlated with (a) age ≤ 10 years; tumor progression for less than 6 months; single tumor location; moderate tumor vascularization; tumor mass size less than 3 cm; no lymph node involvement; no metastasis; no recurrence; clinical staging I or II; and animals that were alive at the end of the followup period. Higher IL-4 protein levels correlated with (b) with age ≤ 10 years; tumor progression for less than 6 months; moderate tumor vascularization; tumor mass size less than 3 cm; no metastasis; no recurrence; clinical staging I or II; and animals that were alive at the end of the followup period. Error bars ± standard error; ^*∗*^ *p* < 0.05; ^*∗∗*^ *p* < 0.01; ^*∗∗∗*^ *p* < 0.001 indicate significant differences according to Student's *t*-test.

*IL-4* was significantly overexpressed in animals aged ≤ 10 years (*p* = 0.02) and animals with tumor time course of less than six months (*p* = 0.01); single tumor location (*p* = 0.0001); moderate tumor vascularization (*p* = 0.0002); tumor mass size less than three centimeters (*p* = 0.0006); no lymph node involvement (*p* = 0.001); metastasis (*p* < 0.0001); recurrence (*p* = 0.0003); and clinical stage I or II (*p* < 0.0001) and animals that were still alive at the end of the followup period (*p* < 0.0001) (Figure 2(a)).

**Figure 3:**
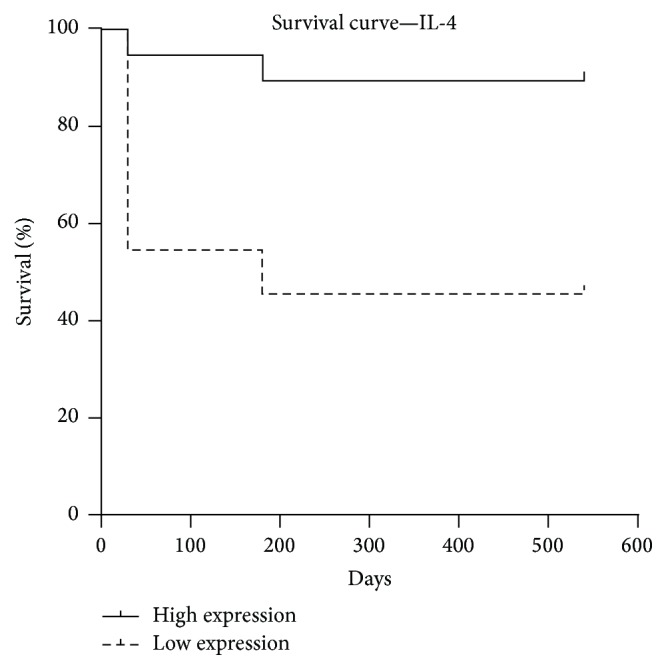
Survival curve of IL-4. Tumors of female dogs with high expression (black line) of IL-4 had lower chance of death compared with animals with low expression (dotted line). *p* = 0.007.

Therefore, IL-4 protein expression was significantly higher in animals aged ≤ 10 years (*p* < 0.0001) and animals with tumor time course of less than six months (*p* = 0.04); moderate tumor vascularization (*p* = 0.03); tumor mass size of less than three centimeters (*p* = 0.001); no metastasis (*p* = 0.01); no recurrence (*p* = 0.02); and clinical stage I or II (*p* = 0.008) and animals that were still alive at the end of the followup period (*p* = 0.005) (Figure 2(b)). Using the ROC curve, the best cutoff point for IL-4 to discriminate the high risk of death was 368.4 pg/mL (sensitivity (95% CI) = 88%, specificity (95% CI) = 95%). The Kaplan-Meier test demonstrated a positive correlation between IL-4 level and survival time (OR 0.068184; 95% CI = 0.783029 to 1; *p* = 0.007) (Figure 3).

**Figure 4:**
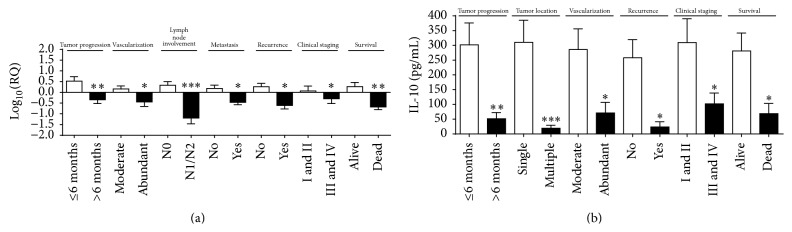
*IL-10* gene overexpression correlated with (a) tumor progression for less than 6 months; moderate tumor vascularization; no lymph node involvement; no metastasis; no recurrence; clinical staging I or II; and animals that were alive at the end of the followup period. Higher IL-10 protein levels correlated with (b) tumor progression for less than 6 months; single tumor location; moderate tumor vascularization; no recurrence; clinical staging I or II; animals that were alive at the end of the followup period. Error bars ± standard error; ^*∗*^ *p* < 0.05; ^*∗∗*^ *p* < 0.01; ^*∗∗∗*^ *p* < 0.001 indicate significant differences according to Student's *t*-test.

For* IL-10*, significant gene overexpression was also correlated with tumor time course of less than six months (*p* = 0.003); moderate tumor vascularization (*p* = 0.02); no lymph node involvement (*p* = 0.0005); no metastasis (*p* = 0.01); no recurrence (*p* = 0.01); clinical stage I or II (*p* = 0.02); and animals that were still alive at the end of the followup period (*p* = 0.009) (Figure 4(a)).

Likewise, IL-10 protein expression was significantly higher in animals with tumor time course of less than six months (*p* = 0.003); single tumor location (*p* = 0.0009); moderate vascularization (*p* = 0.04); no recurrence (*p* = 0.04), and clinical stage I or II (*p* = 0.03) and animals that were still alive during the followup (*p* = 0.04) (Figure 4(b)). For the ROC curve for IL-10, the best cutoff point to discriminate the high risk of death was 243 pg/mL (sensitivity (95% CI) = 100%, specificity (95% CI) = 53%). No correlation was observed between IL-10 protein expression and survival time (OR 0.3267; 95% CI = 0.06811 to 1.564; *p* = 0.16) (data not shown).

**Figure 5:**
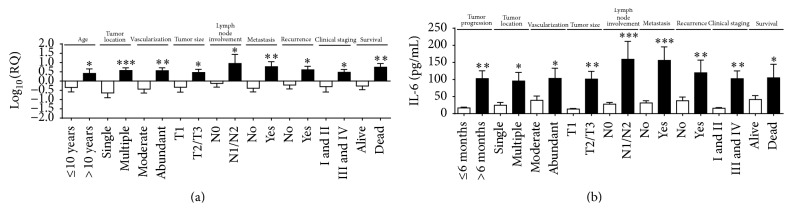
*IL-6* gene overexpression correlated with (a) age ≤ 10 years; multiple tumor locations; abundant tumor vascularization; tumor mass size greater than 3 cm; lymph node involvement; metastasis; recurrence; clinical staging III or IV; and animals that died. Higher IL-6 protein levels correlated with (b) tumor progression for more than 6 months; multiple tumor locations; abundant tumor vascularization; tumor mass size greater than 3 cm; lymph node involvement; metastasis; recurrence; clinical staging III or IV; and animals that died. Error bars ± standard error; ^*∗*^ *p* < 0.05; ^*∗∗*^ *p* < 0.01; ^*∗∗∗*^ *p* < 0.001 indicate significant differences according to Student's *t*-test.

In contrast,* IL-6* was significantly overexpressed in animals aged > 10 years (*p* = 0.03) and animals with multiple tumor locations (*p* = 0.0005); abundant tumor vascularization (*p* = 0.003); tumor mass size greater than three centimeters (*p* = 0.01); lymph node involvement (*p* = 0.04); metastasis (*p* = 0.003); recurrence (*p* = 0.03); and clinical stage III or IV (*p* = 0.02) and animals that died during followup (*p* = 0.008) (Figure 5(a)).

Similarly, IL-6 protein expression was significantly higher in animals with tumor time course greater than six months (*p* = 0.001); multiple tumor locations (*p* = 0.01); abundant vascularization (*p* = 0.02); tumor mass size greater than three centimeters (*p* = 0.001); lymph node involvement (*p* < 0.0001); metastasis (*p* < 0.0001); recurrence (*p* = 0.007); and clinical stage III or IV (*p* = 0.001) and animals that died during followup (*p* = 0.04) (Figure 5(b)). The best cutoff point for IL-6 established by the ROC curve to discriminate the high risk of death was 76.95 pg/mL (sensitivity (95% CI) = 75%, specificity (95% CI) = 86%). No correlation was observed between IL-6 protein expression and survival time (OR 1.719; 95% CI = 0.4045 to 9.580; *p* = 0.43) (data not shown).

**Figure 6:**
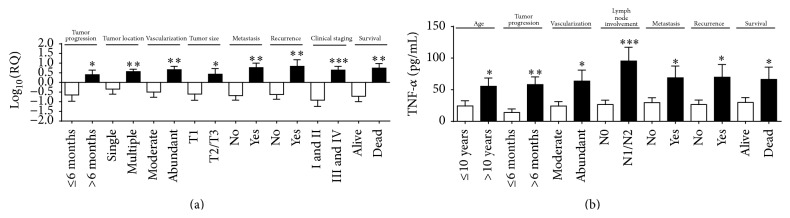
*TNF-α* gene overexpression correlated with (a) tumor progression for more than 6 months; multiple tumor locations; abundant tumor vascularization; tumor mass size greater than 3 cm; metastasis; recurrence; clinical staging III or IV; and animals that died. Higher TNF-*α* protein levels correlated with (b) age > 10 years; tumor progression for more than 6 months; abundant tumor vascularization; lymph node involvement; metastasis; recurrence; and animals that died (*p* = 0.04). Error bars ± standard error; ^*∗*^ *p* < 0.05; ^*∗∗*^ *p* < 0.01; ^*∗∗∗*^ *p* < 0.001 indicate significant differences according to Student's *t*-test.

*TNF-α* was also overexpressed in animals with tumor time course greater than six months (*p* = 0.01); multiple tumor locations (*p* = 0.005); abundant tumor vascularization (*p* = 0.007); tumor mass size greater than three centimeters (*p* = 0.02); metastasis (*p* = 0.001); recurrence (*p* = 0.003); and clinical stage III or IV (*p* = 0.0002) and animals that died during followup (*p* = 0.005) (Figure 6(a)).

**Figure 7:**
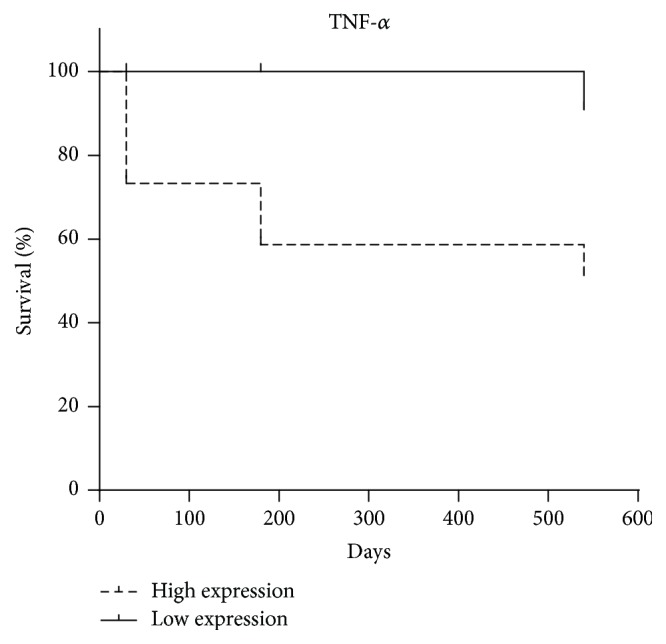
Survival curve of TNF-*α*. Tumors of female dogs with high expression (dotted line) of TNF-*α* had higher chance of death compared with animals with low expression (black line). *p* = 0.01.

The protein expression of TNF-*α* was significantly higher in animals aged > 10 years (*p* = 0.04) and animals with tumor time course greater than six months (*p* = 0.004); abundant vascularization (*p* = 0.01); lymph node involvement (*p* = 0.0007); metastasis (*p* = 0.02); and recurrence (*p* = 0.01) and animals that died during followup (*p* = 0.04) (Figure 6(b)). The best cutoff point to discriminate the high risk of death established by the ROC curve was 16.3 pg/mL (sensitivity (95% CI) = 87%, specificity (95% CI) = 62%). This analysis demonstrated a negative correlation between TNF-*α* protein expression and survival time (OR 5.906; 95% CI = 1.387 to 25.6; *p* = 0.01) (Figure 7).

### 3.2. Spearman's Rank Correlation Analysis between Proinflammatory and Anti-Inflammatory Cytokines with* NF-κB*

**Figure 8:**
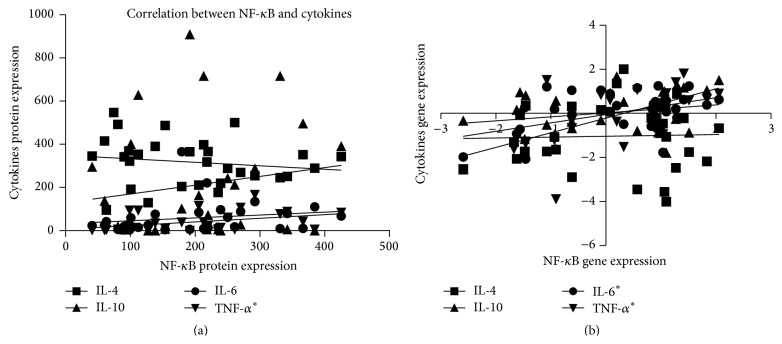
Spearman's rank correlation analysis between proinflammatory and anti-inflammatory cytokines with NF-*κ*B protein and gene expression: (a) positive correlation between NF-*κ*B protein expression and the proinflammatory TNF-*α* cytokine protein expression (*r* = 0.5233; *p* = 0.003) and (b) NF-*κ*B gene expression and its correlation between the proinflammatory TNF-*α* cytokine (*r* = 0.6645; *p* < 0.0001) and IL-6 (*r* = 0.4012; *p* = 0.02) gene expression. ^*∗*^ *p* < 0.05.

Spearman's rank correlation demonstrated that proinflammatory cytokines IL-6 and TNF-*α* are mediated by NF-*κ*B. There was a positive correlation between the proinflammatory TNF-*α* protein and gene expression when compared to NF-*κ*B protein and gene expression (*r* = 0.5233, *p* = 0.003; *r* = 0.6645, *p* < 0.0001; Figures 8(a) and 8(b), resp.). In the same way, there was a positive correlation between IL-6 and NF-*κ*B gene expression (*r* = 0.4012; *p* = 0.02) (Figure 8(b)). Furthermore, the mutual balance between proinflammatory cytokines and anti-inflammatory ones was analyzed by Spearman's rank correlation and showed no correlation (*p* > 0.05; data not shown).

### 3.3. Multivariate Analysis

The multivariate analysis of high levels of the proinflammatory cytokines, low levels of the anti-inflammatory cytokines, and clinicopathological features of poor prognosis with regard to an increased risk of death showed no correlation (*p* > 0.05) (Table 2).

## 4. Discussion

NF-*κ*B is a crucial factor between chronic inflammation and cancer development [34] and it regulates both anti- and proinflammatory cytokines at various stages of tumorigenesis and inflammation [35, 36]. We observed high gene and protein expression levels of NF-*κ*B in animals with tumor progression for greater than six months, multiple tumor locations, abundant vascularization, and metastasis, indicating a role of this factor in angiogenesis support and inhibition of apoptosis in tumor cells. In accordance with our prediction, Kiliccioglu et al. [37] observed that NF-*κ*B inhibition, through proteasome inhibition, induces apoptosis in human prostate cancer cell line (PC3), resulting in significantly increased protein levels of caspase-3, which plays a central role in the execution phase of cell apoptosis. Similarly, Chien et al. [38] reported that pancreatic cancer cell growth is suppressed by inhibiting the NF-*κ*B pathway.

In the same way, high protein levels of NF-*κ*B were observed in female dogs above 10 years old which could indicate the correlation between this nuclear factor and cell oxidative stress, leading to tissue damage and chronic inflammation. Corroborating with this, a review of oxidative stress and breast cancer, recently published by Nourazarian et al. [39], suggests that the increase of free radical levels in tumor cells occurs under the influence of the increased expression of cellular enzymes and enhanced activity of some dependent tumor cells, such as cancer-associated fibroblasts (CAFs) and tumor-associated macrophages (TAMs) [39, 40].

Once NF-*κ*B has been activated through one of many pathways, crucial properties of the malignant phenotype are activated, promoting tumor cell adaptation to the tumor environment [41]; then, we also reported* NF-κB* overexpressed in female dogs with tumor masses greater than three centimeters, lymph node involvement, recurrence, and clinical stage III or IV, leading to a poor survival rate of the animals.

It is known that NF-*κ*B is required for the expression of many cytokine genes that encode proteins associated with inflammatory mediators [42]. Then, through Spearman's rank correlation, we showed that high expression of NF-*κ*B modulates positively the IL-6 and TNF-*α* expression levels in mammary tumors. The proinflammatory cytokines IL-1*β*, IL-6, and TNF-*α* could be modulated by lipopolysaccharides (LPS) administration as it occurs in inflammatory diseases. According to Cheon et al. [43], some drugs, as saxifragin, inhibited the LPS-induced nuclear translocation of p65 and the activation of caspase-1 in RAW 264 cells, suggesting that the NF-*κ*B-regulated gene transcription is responsible for anti-inflammatory response of macrophages. In addition, Osnes et al. [44] affirmed that the inhibitory synthesis of TNF-*α* by drugs as acetylsalicylic acid and sodium salicylate in human monocytes is due to interfering of nuclear translocation of NF-*κ*B/c-Rel proteins.

Besides that, corroborating with our study, Al-Halabi et al. [34] showed that the inhibition of NF-*κ*B significantly lowers microvessel density (CD31) and mRNA expression levels of IL-6, TNF-*α*, and IL-1*α*, as well as protein levels of proliferation marker (Ki-67) and vascular endothelial growth factor- (VEGF-) A in human colon cancer, confirming the primordial role of NF-*κ*B in angiogenesis and, therefore, tumor progression.

Regarding anti-inflammatory interleukins, our study sustains a protector action of the anti-inflammatory cytokines IL-4 and IL-10 in female dogs' mammary tumors. IL-4 was highly expressed in gene and protein levels in female dogs with a better prognostic. This cytokine acts on endothelial cells, inhibiting tumor-induced vascularization and starving tumor cells [45], and also induces secretion of IL-10, the Th2 cytokine with a strong suppressive effect on tumors [46, 47], which could explain high levels of this cytokine in animals that are still alive after the followup period and in overall survival. Furthermore, according to Okada and Kuwashima [45], the sustained expression of IL-4 may provide effective means for therapy of a variety of diseases, including cancer.

In addition, high gene and protein expression of IL-10 were correlated with characteristics of better prognosis. IL-10 downregulates proinflammatory cytokine expression [48, 49] and inhibits the expression of CD31 [50]. Lin and Karin [48] reported that IL-10 could modulate apoptosis and suppress angiogenesis during tumor regression, downregulating VEGF, TNF-*α*, and IL-6 production by TAMs. Jindal and Borges [51] showed that IL-10 inhibits tumor growth by preventing chemokine expression and angiogenesis when administered at higher doses in cancer animal models. Li et al. [52] correlated high IL-10 expression in female dogs with no metastasis or recurrence and high overall survival. Contrarily, gene overexpression of* IL-10* in animals that had metastasis could suggest posttranscriptional regulation of this cytokine, once the protein levels do not show this correlation.

IL-6 mediates a plethora of physiological functions, including the developmental differentiation of lymphocytes, cell proliferation, and cell survival during tumorigenesis [48, 53]. This cytokine had high gene and protein expression correlated with poor prognosis and a negative correlation was found in animals that died during the followup. In the same way, a study by Madeddu et al. [54] demonstrated high expression of IL-6 through activation of Akt/PI3K/mTOR cascade due to elevated oxidative stress, promoting oncogenesis and tumor progression. Owing to its association with poor prognosis, researchers have proposed IL-6 as a therapeutic target in cancer [49]. Several phase I/II clinical trials are currently evaluating antibodies against IL-6 or IL-6R as therapeutic alternatives for prostate cancer [53, 55] and renal cancer [56, 57]. These results suggest that further studies are needed to determine the appropriate use of anti-interleukin monoclonal antibodies as a therapeutic treatment, and studies similar to ours, evaluating interleukin expression, may be useful as a foundation for further clinical trials of therapy using cytokines.

TNF-*α* is a cytokine that promotes tumorigenesis under conditions of unresolved inflammation [49]. Increasing lines of evidence suggest that TNF-*α* regulates many of the critical processes of tumor promotion and progression [48, 58]. In our study, high protein levels of TNF-*α* were found in animals that died during the followup and correlated with worse prognostic features. Its cytokine has been implicated yet as a tumor promoter in different tumor types like ovarian cancer [59], gallbladder cancer [60], and oral squamous cell carcinoma [61]. TNF-*α* binds to two receptors, namely, the ubiquitously expressed TNF receptor 1 (TNFR1) and hematopoietically restricted TNFR2, and modulates a signaling cascade that induces mediators of transcriptional regulation that are key to cell survival, invasion, angiogenesis, and impairment of immune surveillance in tumor biology [49, 58, 59, 62].

Altogether, our data showed the effect of cytokines on tumor development and progression, as well as positive modulation of NF-*κ*B and proinflammatory interleukins in mammary tumors. Analysis of the active genes and proteins in cancer can be used to target treatment more specifically, and our results suggest the use of these biological factors as prognostic biomarkers in cancer.

## 5. Conclusion

Our results are of utmost importance for a better understanding of the dynamic network of cytokines and the influence of NF-*κ*B on tumorigenesis. These cytokines could be employed as noninvasive prognostic markers in female dogs with mammary tumors and could be useful for predicting disease progression and tumor recurrence. There is lack of studies devoted to diagnostic, therapeutic, and prognostic markers in canine mammary tumors; so, this study defines potential prognostic and predictive markers for routine use in the clinic, allowing the detection of recurrence and metastasis, which could allow the early adoption of more precise conduct to improve the overall survival of animals.

## Figures and Tables

**Table 1:** Clinicopathological parameters of female dogs with mammary tumors.

Clinicopathological parameters	Number of dogs
Age of animals	
>10 years	14 (53%)
≤10 years	16 (47%)
Tumor time course	
>six months	16 (54%)
≤six months	14 (44%)
Tumor location	
Multiple	14 (44%)
Single	16 (54%)
Vascularization	
Moderate	20 (66%)
Abundant	10 (34%)
Tumor mass size	
T1	15 (50%)
T2/T3	15 (50%)
Lymph node involvement	
N0	25 (84%)
N1/N2	5 (16%)
Metastasis	
Yes	8 (27%)
No	22 (73%)
Clinical staging	
I and II	14 (44%)
III and IV	16 (54%)
Recurrence	
Yes	8 (27%)
No	22 (73%)
Censorship	
Death	8 (27%)
Alive	22 (73%)

**Table 2:** Multivariate analysis of proinflammatory and anti-inflammatory cytokines and clinicopathological features of poor prognosis with regard to an increased risk of death.

Cytokines	OD	95% CI	*p* value
IL-4	7.819735	0.48417 to 126.294945	0.1
IL-6	1.3169	0.09401 to 18.447342	0.8
IL-10	0.199689	0.01511 to 2.639014	0.2
TNF-*α*	0.259712	0.011486 to 5.872229	0.3

## References

[1] Lorusso G., Rüegg C. (2008). The tumor microenvironment and its contribution to tumor evolution toward metastasis. *Histochemistry and Cell Biology*.

[2] Leonardi G. C., Candido S., Cervello M. (2012). The tumor microenvironment in hepatocellular carcinoma (review). *International Journal of Oncology*.

[3] Tepper R. I., Coffman R. L., Leder P. (1992). An eosinophil-dependent mechanism for the antitumor effect of interleukin-4. *Science*.

[4] Lu C., Sheehan C., Rak J. W., Chambers C. A., Hozumi N., Kerbel R. S. (1996). Endogenous interleukin 6 can function as an in vivo growth-stimulatory factor for advanced-stage human melanoma cells. *Clinical Cancer Research*.

[5] Kobayashi M., Kobayashi H., Pollard R. B., Suzuki F. (1998). A pathogenic role of Th2 cells and their cytokine products on the pulmonary metastasis of murine B16 melanoma. *Journal of Immunology*.

[6] Benchetrit F., Ciree A., Vives V. (2002). Interleukin-17 inhibits tumor cell growth by means of a T-cell-dependent mechanism. *Blood*.

[7] Hamidullah, Changkija B., Konwar R. (2012). Role of interleukin-10 in breast cancer. *Breast Cancer Research and Treatment*.

[8] Karin M. (2006). Nuclear factor-*κ*B in cancer development and progression. *Nature*.

[9] Naugler W. E., Karin M. (2008). NF-*κ*B and cancer-identifying targets and mechanisms. *Current Opinion in Genetics and Development*.

[10] Beg A. A., Baltimore D. (1996). An essential role for NF-*κ*B in preventing TNF-*α*-induced cell death. *Science*.

[11] Biswas D. K., Martin K. J., McAlister C. (2003). Apoptosis caused by chemotherapeutic inhibition of nuclear factor-*κ*B activation. *Cancer Research*.

[12] Gelaleti G. B., Jardim B. V., Leonel C., Moschetta M. G., Zuccari D. A. P. D. C. (2012). Interleukin-8 as a prognostic serum marker in canine mammary gland neoplasias. *Veterinary Immunology and Immunopathology*.

[13] Wilczyński J. R. (2006). Cancer and pregnancy share similar mechanisms of immunological escape. *Chemotherapy*.

[14] Nicolini A., Carpi A., Rossi G. (2006). Cytokines in breast cancer. *Cytokine and Growth Factor Reviews*.

[15] Saleh M., Davis I. D., Wilks A. F. (1997). The paracrine role of tumour-derived mIL-4 on tumour-associated endothelium. *International Journal of Cancer*.

[16] Gooch J. L., Lee A. V., Yee D. (1998). Interleukin 4 inhibits growth and induces apoptosis in human breast cancer cells. *Cancer Research*.

[17] Gomes M., Coelho A., Araújo A., Teixeira A. L., Catarino R., Medeiros R. (2012). Influence of functional genetic polymorphism (− 590C/T) in non-small cell lung cancer (NSCLC) development: the paradoxal role of IL-4. *Gene*.

[18] Gooch J. L., Christy B., Yee D. (2002). STAT6 mediates interleukin-4 growth inhibition in human breast cancer cells. *Neoplasia*.

[19] O'Garra A., Vieira P. (2007). TH1 cells control themselves by producing interleukin-10. *Nature Reviews Immunology*.

[20] Heckel M. C., Wolfson A., Slachta C. A. (2011). Human breast tumor cells express IL-10 and IL-12p40 transcripts and proteins, but do not produce IL-12p70. *Cellular Immunology*.

[21] Lentsch A. B., Shanley T. P., Sarma V., Ward P. A. (1997). In vivo suppression of NF-kappa B and preservation of I kappa B alpha by interleukin-10 and interleukin-13. *The Journal of Clinical Investigation*.

[22] Mumm J. B., Emmerich J., Zhang X. (2011). IL-10 elicits IFN*γ*-dependent tumor immune surveillance. *Cancer Cell*.

[23] Drygin D., Ho C. B., Omori M. (2011). Protein kinase CK2 modulates IL-6 expression in inflammatory breast cancer. *Biochemical and Biophysical Research Communications*.

[24] Germano G., Allavena P., Mantovani A. (2008). Cytokines as a key component of cancer-related inflammation. *Cytokine*.

[25] Le Bitoux M.-A., Stamenkovic I. (2008). Tumor-host interactions: the role of inflammation. *Histochemistry and Cell Biology*.

[26] Kim S., Keku T. O., Martin C. (2008). Circulating levels of inflammatory cytokines and risk of colorectal adenomas. *Cancer Research*.

[27] Soria G., Ofri-Shahak M., Haas I. (2011). Inflammatory mediators in breast cancer: coordinated expression of TNF*α* & IL-1*β* with CCL2 & CCL5 and effects on epithelial-to-mesenchymal transition. *BMC Cancer*.

[28] Madhusudan S., Muthuramalingam S. R., Braybrooke J. P. (2005). Study of etanercept, a tumor necrosis factor-alpha inhibitor, in recurrent ovarian cancer. *Journal of Clinical Oncology*.

[29] Tang F., Tang G., Xiang J., Dai Q., Rosner M. R., Lin A. (2002). The absence of NF-*κ*B-mediated inhibition of c-Jun N-terminal kinase activation contributes to tumor necrosis factor alpha-induced apoptosis. *Molecular and Cellular Biology*.

[30] Kawaguchi M., Adachi M., Oda N., Kokubu F., Huang S.-K. (2004). IL-17 cytokine family. *Journal of Allergy and Clinical Immunology*.

[31] Misdorp W., Else R. W., Hellmén E., Lipscomb E. (1999). Definitions and explanatory notes. *Who Histological Classification of Mammary Tumors of the Dog and Cat*.

[32] Cassali G. D., Bertagnolli A. C., Lavalle G. E., Tavares W. L. F., Ferreira E., Silva A. E. Perspectives for diagnosis, prognosis and treatment of mammary neoplasms in dogs.

[33] Schmittgen T. D., Livak K. J. (2008). Analyzing real-time PCR data by the comparative **C**
_T_ method. *Nature Protocols*.

[34] Al-Halabi R., Bou Chedid M., Abou Merhi R. (2011). Gallotannin inhibits NF*κ*B signaling and growth of human colon cancer xenografts. *Cancer Biology and Therapy*.

[35] Gupta S. C., Sundaram C., Reuter S., Aggarwal B. B. (2010). Inhibiting NF-*κ*B activation by small molecules as a therapeutic strategy. *Biochimica et Biophysica Acta*.

[36] Del Prete A., Allavena P., Santoro G., Fumarulo R., Corsi M. M., Mantovani A. (2011). Molecular pathways in cancer-related inflammation. *Biochemia Medica*.

[37] Kiliccioglu I., Konac E., Varol N., Gurocak S., Yucel Bilen C. (2014). Apoptotic effects of proteasome and histone deacetylase inhibitors in prostate cancer cell lines. *Genetics and Molecular Research*.

[38] Chien W., Lee D. H., Zheng Y. (2014). Growth inhibition of pancreatic cancer cells by histone deacetylase inhibitor belinostat through suppression of multiple pathways including HIF, NFkB, and mTOR signaling in vitro and in vivo. *Molecular Carcinogenesis*.

[39] Nourazarian A. R., Kangari P., Salmaninejad A. (2014). Roles of oxidative stress in the development and progression of breast cancer. *Asian Pacific Journal of Cancer Prevention*.

[40] Sosa V., Moliné T., Somoza R., Paciucci R., Kondoh H., LLeonart M. E. (2013). Oxidative stress and cancer: an overview. *Ageing Research Reviews*.

[41] Tafani M., Pucci B., Russo A. (2013). Modulators of HIF1*α* and NFkB in cancer treatment: is it a rational approach for controlling malignant progression?. *Frontiers in Pharmacology*.

[42] Baldwin A. S. (2001). The transcription factor NF-*κ*B and human disease. *Journal of Clinical Investigation*.

[43] Cheon S. Y., Chung K. S., Jeon E., Nugroho A., Park H. J., An H. J. (2015). Anti-inflammatory activity of saxifragin via inhibition of NF-*κ*B involves caspase-1 activation. *Journal of Natural Products*.

[44] Osnes L. T. N., Foss K. B., Joo G. B. (1996). Acetylsalicylic acid and sodium salicylate inhibit LPS-induced NF-kappa B/c-Rel nuclear translocation, and synthesis of tissue factor (TF) and tumor necrosis factor alfa (TNF-alpha) in human monocytes. *Thrombosis and Haemostasis*.

[45] Okada H., Kuwashima N. (2002). Gene therapy and biologic therapy with interleukin-4. *Current Gene Therapy*.

[46] Li Z., Chen L., Qin Z. (2009). Paradoxical roles of IL-4 in tumor immunity. *Cellular and Molecular Immunology*.

[47] Pericle F., Giovarelli M., Colombo M. P. (1994). An efficient Th2-type memory follows CD8+ lymphocyte-driven and eosinophil-mediated rejection of a spontaneous mouse mammary adenocarcinoma engineered to release IL-4. *Journal of Immunology*.

[48] Lin W.-W., Karin M. (2007). A cytokine-mediated link between innate immunity, inflammation, and cancer. *Journal of Clinical Investigation*.

[49] Landskron G., De la Fuente M., Thuwajit P., Thuwajit C., Hermoso M. A. (2014). Chronic inflammation and cytokines in the tumor microenvironment. *Journal of Immunology Research*.

[50] Shi J., Li J., Guan H. (2014). Anti-fibrotic actions of interleukin-10 against hypertrophic scarring by activation of PI3K/AKT and STAT3 signaling pathways in scar-forming fibroblasts. *PLoS ONE*.

[51] Jindal S., Borges V. F. (2011). The emerging role of cytokines in breast cancer: from initiation to survivorship. *Breast Cancer*.

[52] Li Y., Yu H., Jiao S., Yang J. (2014). Prognostic value of IL-10 expression in tumor tissues of breast cancer patients. *Xi Bao Yu Fen Zi Mian Yi Xue Za Zhi*.

[53] Karkera J., Steiner H., Li W. (2011). The anti-interleukin-6 antibody siltuximab down-regulates genes implicated in tumorigenesis in prostate cancer patients from a phase I study. *Prostate*.

[54] Madeddu C., Gramignano G., Floris C., Murenu G., Sollai G., Macciò A. (2014). Role of inflammation and oxidative stress in post-menopausal oestrogen-dependent breast cancer. *Journal of Cellular and Molecular Medicine*.

[55] Fizazi K., De Bono J. S., Flechon A. (2012). Randomised phase II study of siltuximab (CNTO 328), an anti-IL-6 monoclonal antibody, in combination with mitoxantrone/prednisone versus mitoxantrone/prednisone alone in metastatic castration-resistant prostate cancer. *European Journal of Cancer*.

[56] Puchalski T., Prabhakar U., Jiao Q., Berns B., Davis H. M. (2010). Pharmacokinetic and pharmacodynamic modeling of an anti-interleukin-6 chimeric monoclonal antibody (siltuximab) in patients with metastatic renal cell carcinoma. *Clinical Cancer Research*.

[57] Rossi J.-F., Négrier S., James N. D. (2010). A phase I/II study of siltuximab (CNTO 328), an anti-interleukin-6 monoclonal antibody, in metastatic renal cell cancer. *British Journal of Cancer*.

[58] Szlosarek P., Charles K. A., Balkwill F. R. (2006). Tumour necrosis factor-*α* as a tumour promoter. *European Journal of Cancer*.

[59] Charles K. A., Kulbe H., Soper R. (2009). The tumor-promoting actions of TNF-*α* involve TNFR1 and IL-17 in ovarian cancer in mice and humans. *Journal of Clinical Investigation*.

[60] Zhu G., Du Q., Wang X., Tang N., She F., Chen Y. (2014). TNF-*α* promotes gallbladder cancer cell growth and invasion through autocrine mechanisms. *International Journal of Molecular Medicine*.

[61] Lee S. H., Hong H. S., Liu Z. X. (2012). TNF*α* enhances cancer stem cell-like phenotype via Notch-Hes1 activation in oral squamous cell carcinoma cells. *Biochemical and Biophysical Research Communications*.

[62] Balkwill F. (2006). TNF-*α* in promotion and progression of cancer. *Cancer and Metastasis Reviews*.

